# Biobehavioral survey using time location sampling among female sex workers living in Ghana in 2020

**DOI:** 10.3389/fpubh.2024.1137799

**Published:** 2024-02-16

**Authors:** Samuel Dery, Chris Guure, Seth Afagbedzi, Augustine Ankomah, William Ampofo, Kyeremeh Atuahene, Comfort Asamoah-Adu, Ernest Kenu, Sharon Stucker Weir, Waimar Tun, Daniel Arhinful, Kwasi Torpey

**Affiliations:** ^1^Department of Biostatistics, School of Public Health, University of Ghana, Accra, Ghana; ^2^Population Council, Accra, Ghana; ^3^University of Ghana Noguchi Memorial Institute for Medical Research, Accra, Ghana; ^4^Ghana AIDS Commission, Accra, Ghana; ^5^West Africa Program to Combat AIDS and STI (WAPCAS), Accra, Ghana; ^6^Department of Epidemiology and Disease Control, School of Public Health, University of Ghana, Accra, Ghana; ^7^Department of Epidemiology, University of North Carolina, Chapel Hill, NC, United States; ^8^HIV and AIDS Program, Population Council, Washington, DC, United States; ^9^Department of Population, Family and Reproductive Health, School of Public Health, University of Ghana, Accra, Ghana

**Keywords:** female sex workers, commercial sex workers, roamer population, seater population, HIV, STIs, time location sampling, venue-day time sampling

## Abstract

**Background:**

The HIV epidemic in Ghana is characterized as a mix of a low-level generalized epidemic with significant contributions from transmission among female sex workers (FSW) and their clients. This study seeks to identify and describe key characteristics and sexual behaviors of FSW and estimate the prevalence of HIV, syphilis, gonorrhea, chlamydia, and hepatitis B virus (HBV) among FSW in Ghana.

**Method:**

A total of 7,000 FSW were recruited for the study using Time Location Sampling (TLS) approach with 5,990 (85.6%) participants completing both biological and the behavioral aspects of the study. A structured questionnaire was administered to respondents to assess several factors, such as background characteristics, sexual risk behaviors, condom usage, HIV/AIDS knowledge, opinions, and attitudes. Trained staff conducted face-to-face interviews using mobile data collection software (REDCap) after provision of specimens for HIV and STI testing. Descriptive statistics such as medians, ranges, charts, and percentages are performed and presented. Also included, are bivariate analyses to establish relationships between FSW type and other relevant characteristics of the study.

**Results:**

Among the 7,000 (100%) FSW sampled from all regions, 6,773 took part in the behavioral and 6,217 the biological. There were 783 (11.2%) respondents who took part only in the behavioral and 227 (3.2%) only in the biological. Most were young, with a median age of 26 years, majority had never been married or were widowed/divorced and a quarter had no education or had only primary education. Majority (74.8%) of FSW first sold sex at age 25 years or less with a median age of 20 years. Most (84.8%) of the FSW indicated that they entered sex work for money, either for self or family and had an average of eleven (11) sexual partners per week. More than half (55.2%) of the FSW were new entrants who had been in sex work for less than 5 years before the study. Consistent condom use with paying clients was generally unsatisfactory (71%), and was however, very low (24%) with their intimate partners or boyfriends. Only about half (54.6%) of FSW have been exposed to HIV prevention services in the last three months preceding the survey, and this varies across regions. Overall, comprehensive knowledge about HIV and AIDS was low. Only 35% of FSW had comprehensive knowledge. HIV prevalence was 4.6% and was higher among seaters (brothel-based) and older FSW who had been sex work for a longer period. The HIV prevalence from the previous bio-behavioral survey (BBS) in 2015 and 2011 were estimated to be 6.9 and 11.1%, respectively.

**Conclusion:**

Compared to the results from the previous studies, the findings give an indication that Ghana is making significant progress in reducing the burden of HIV among FSW in the country. However, risky behaviors such as low consistent condom use, low coverage of HIV services across the regions, and low comprehensive knowledge could reverse the gains made so far. Immediate actions should be taken to expand coverage of HIV services to all locations. Efforts must be made to reach out to the new entrants while also addressing strongly held myths and misconceptions about HIV.

## Background

Ghana’s HIV epidemic has over the years been described as generalized epidemic with prevalence of more than 1 % in the general population. According to the 2014 Ghana demographic and health survey (GDHS), HIV prevalence in the country was 2.0%, having decreased marginally from 2.2% in 2006 (1). In addition, HIV prevalence from the national estimates have been declining over the years from 1.87% in 2010 to 1.70% in 2019 and the estimated new infections also decreased from 23,199 in 2010 to 20,068 in 2019 (2). The 2014 modes of transmission (MoT) study revealed that female sex workers (FSW) and men who have sex with men (MSM) contribute significantly to the HIV epidemic in the country (3). Though commercial sex work in Ghana is illegal, sex work activities are still being practiced across the various regions in the country with high HIV prevalence among female sex workers (4).

The 2015 bio-behavioral survey (BBS) among FSW showed the HIV prevalence was 7.0% (5). Even though this prevalence is still unacceptably high compared to the general population, there has been consistent decrease in HIV prevalence among FSW over the past 15 years, from 34% in 2006, 25.1% in 2009 to 11.1% in 2011 (6). There exist also, regional differences in the HIV prevalence among FSW in the country. Evidence from the 2014 modes of transmission (MoT) study show sex work accounted for 18.4% of all new infections at the national level in 2014, having declined from 27% in 2009 (3). In response to these gaps, Ghana developed the National HIV and AIDS Strategic Plan (NSP 2016–2020) to fast track the country’s effort toward ending AIDS by 2030. The plan prioritized prevention, treatment, and care programs for key populations (KPs), as it sought to reduce HIV prevalence among FSW to 5.6% in 2020 (10.7% for seaters and 3.4% for roamers) (7). Though there are no regulation regarding HIV testing among FSW, HIV prevention interventions recommend regular testing (every six months) among FSW in Ghana.

Despite these findings from the previous BBS and the MoT study, there continue to be limited up-to-date information on characteristics, health-seeking, and HIV risk behaviors among FSW, thereby, hindering the ability to design and implement appropriate HIV interventions for FSW. The national HIV and AIDS monitoring and evaluation plan recommends that biological and behavioral surveys (BBS) among KPs should be done every 3 to 4 years (8).

Various typologies have been used to classify sex workers based on the main places of solicitation and identified typologies such as: brothel-based, home-based, street-based, and other FSW (9, 10). In a systematic review by Pitpitan et al., they found that sex work typology was useful in differentiating between FSW who had higher versus lower HIV/STI prevalence. They further hypothesized that “HIV risk among FSW is partly a function of venues or types of sex work” (11). A study in Thailand also found a lower HIV prevalence among brothel-based FSW than street-based FSW (12). This suggests that there may be differences in risky behaviors and uptake of HIV prevention interventions by FSW type.

The 2020 round of the FSW BBS is thus a follow up on the previous two rounds carried out in 2011, and 2015. This study sought to describe key characteristics and sexual behaviors of FSW and estimate the prevalence of HIV, syphilis, gonorrhea, chlamydia, and hepatitis B virus (HBV) among FSW, and to compare these characteristics between FSW type (seater or roamer). The findings of the study will serve as an evaluation of the national strategic plan implementation, and eventually inform the development and implementation of the National HIV and AIDS Strategic Plan 2021–2025.

## Methods

### Target populations and geographic areas

This study enrolled FSW in all regions of Ghana. Female Sex Worker was defined in the study as any female aged 16 years or older who reports to have exchanged sexual acts in the last 6 months with someone other than her established partner for something of value (13). This exchange can be either regular or occasional. There were two main categories of FSW, namely: seaters who operate at specific defined/fixed and well-known locations, and roamers who are not associated with a specific defined location but move from one location to another to seek clients.

### Sample size determination

The sample size for both Seaters and Roamers was determined using “recently tested for HIV” indicator (proportion of FSW who said yes to having had an HIV test in the last 3 months preceding the survey) from the 2015 BBS conducted in Ghana (5). The 2015 BBS shows that 48% roamers and 61% seaters indicated that they tested for HIV within the last 3 months preceding the survey. The sample size was calculated based on detecting a change of 12 percentage points for both roamers, and seaters with an alpha of 0.03 and 80% power. A design effect of 1.5 and a non-response rate of 10% were factored in. After adjusting for strata [total number of regions (10) in Ghana at the time], the sample sizes were 4,580 for seaters, and 5,150 for roamers for the whole country. However, based on the BBS 2015, estimated seater FSW population size of 2,319, the Finite Population Correction Formula (FPCF) was used, which yielded a sample size target of 1,540 for seaters. Therefore, the total sample size for the study was 6,690.

### Study design

The study used a cross sectional design approach however, a pre-survey assessment was undertaken in preparation for the main study. The pre-survey activities included national and regional stakeholder consultations of civil society organizations (CSOs) who work with or have worked with FSW in the last two years, as well as the Ghana Police Service. In addition, all the venues where FSW could be found were identified and verified by our trained field staff. Research staff were provided with venues that were identified in the previous two rounds of the BBS in 2011 and 2015. Additionally, CSOs, gatekeepers, the Ghana Police Service provided to the research team, and a list of potential active venues across the country. In all, 207 research assistants and 44 field supervisors were deployed simultaneously across the entire country to verify the existence of the venues and also identify venues that were not included in the list submitted to the team. This exercise took three weeks. All newly identified and verified venues were subsequently mapped using Geographic Information System (GIS) techniques and a Global Positioning System (GPS)/Tablet to collect geographic coordinates. The total number of active FSW venues found and mapped nationwide were 2,482.

### Selection of venues and participants

Time Location Sampling (TLS) was employed to select and interview respondents. It is a probability-based method used to enroll members of a key population that takes into consideration the day, time and location where they congregate. Briefly, TLS is used when the number of people attending a venue varies over time or day. It is sometimes called venue-day time (VDT) sampling. It is defined according to location (venues where FSW congregate) and time (when the majority of FSW congregate) (14, 15). The sampling frame was developed from the list of VDT derived from the sampling universe that was obtained during the mapping.

The data from the mapping and venue verification exercise, the list of all the venues or primary sampling units (Venue, Day and Time – VDT) from the sampling frame (grouped into four-hour intervals for each VDT) were used. In addition, a comprehensive sampling frame for each population group at each venue was developed and organized systematically by key geographic units. This mapping strategy was used to develop the sampling frame for the venues in the regions and at the national level.

A simple random sample of venues was selected from the sampling frame exclusively for unique venues. This was followed by another simple random selection of a specific day and time period associated with the sampled venue. Details of the three stages conducted sequentially are below:

Stage 1: A list of unique venues was compiled without recourse to the day and time specified for each venue. Out of this list, venues were randomly selected. Independent venue selection was conducted by region. Venue selection was done without replacement.

Stage 2: A second layer of simple random sampling was employed to sample one out of the number of VDTs without replacement. This process continued until all venues selected in stage 1 were either exhausted or regional allocation of sample size was reached. In a few instances scheduling conflicts occurred. For example, if two venues were due for the selection, but was not feasible to visit both at the same time, the remaining VDT for that venue was randomly selected to fill that gap. Alternative venues were selected using the same process. In some of the regions, these venues were used to replace a few selected venues that were canceled as a result of non-cooperation on the part of venue owners/managers, or temporary closure, or for security reasons. New venues discovered during the field exercise were added to the sampling frame for the alternative samples.

Stage 3: Upon arrival at each of the VDTs, the field supervisors, with the help of the peer educators, counted the number of women present at the venue. Other women who arrived at the venue after the study team’s arrival, were also counted. At the end of the survey, the total number of women who visited the venue were recorded. Almost all the women found at the venue were approached using a screening log form where the number of FSW approached, screened, eligible, willing/interviewed was recorded. All interviews were conducted within a four (4) hour interval with strict adherence. Some of these values were used to calculate sampling weights to adjust estimates and to calculate refusal rate for the study.

For each venue, nearby private rooms that were quiet and spacious enough to accommodate both the behavioral and the biological teams were acquired for interviews. At these venues, all participants were screened and those eligible were informed of the study and signed the informed consent form to take part in the study. For those who consented, a study card bearing a unique ID was issued to the participant after which a nurse provided pretest counseling and a laboratory technician collected biological samples for testing. The electronic questionnaire in REDCap was then administered by the trained research assistant.

### Data collection tools, techniques, and statistical analysis

In the survey, a structured questionnaire was adapted from the Bio-behavioral Survey Guidelines for Populations at Risk of HIV (14) and the questionnaire used in the previous study. The questionnaire was reviewed by the Scientific Advisory Committee and further modifications were made after the pilot study in 3 regions. The pilot study enabled the team to assess several aspects of the study, including duration for the administration of the questionnaire, and the acceptability and availability of the FSW to take part in the interviews. Their willingness to take part in the biological part of the survey by providing blood and urine samples was also assessed. The questionnaire assessed several factors including background characteristics, sexual risk behaviors, condom usage, knowledge, opinions and attitude regarding HIV and AIDS, stigma and discrimination, exposure to HIV programs, STIs, and injection of drugs among others. Comprehensive knowledge of AIDS was also measured as: (i) knowing that both limiting sex partners to one uninfected partner and consistent condom use are HIV prevention methods, (ii) knowing that a healthy-looking person can have HIV, and (iii) rejecting two of the most common misconceptions – that HIV can be transmitted through mosquito bites and by supernatural means.

An online version of the study questionnaire was designed in Research Electronic Data Capture (REDCap) and uploaded onto a mobile (android and IOS) app that was in sync with the REDCap Server. This made it possible for the research assistants to synchronize data directly to the server. The data was collected from February to March 2020 across all the regions of Ghana.

To obtain regional and national level estimates, sampling weights were calculated and applied to the dataset following the method recommended by University of California San Francisco (UCSF) in TLS guidance, details of the weighting approach is provided elsewhere (16). Briefly, the weight for combining regional data for national estimates was calculated as the inverse of the probability that the FSW was sampled, adjusting for the proportion of venues sampled in the region. Specifically, the national weight was calculated as the inverse of the estimated probability that the FSW was interviewed at the selected venue, times the estimated probability that the venue was selected in the region.

The statistical methods employed included descriptive analysis to obtain data summaries of key indicators and variables and presented mostly in frequencies, proportions, percentages, means/medians where necessary. In addition, bivariate analysis via the Pearson’s chi-squared test statistic, and multivariable logistics regression with the sole aim of establishing whether there is any association or relationship between FSW type (seater or roamer) and other independent variables were employed. In the final model, independent variables were retained only if they were further found to be significant at 5% level to the outcome of interest.

### HIV and STIs testing

Testing of HIV and other STIs was conducted by study staff in line with national guidelines and established study specific protocols. After pre-test counseling, laboratory technicians collected 6 mL of blood and 10 mL of urine from each study participant. First, void urine samples for the detection of *Neisseria gonorrhea* and *Chlamydia trachomatis* were collected from all survey respondents and stored in clean sterile 50 mL polypropylene containers without preservatives. In the field, the samples were stored in a cool box with ice packs. They were then moved to the laboratory of the hub health facility and finally transported in a cool box to the Noguchi Memorial Institute for Medical Research (NMIMR), University of Ghana, for processing. Universal precautions were followed, and samples were labeled with unique study identification numbers.

After onsite testing were completed, blood and urine samples were transported to the health facility laboratory, processed, stored and then shipped with ice packs in cool boxes to the NMIMR, Accra, by courier. Serological and molecular testing for the selected pathogens utilized commercial assays. Laboratory staff performed the tests in accordance with criteria outlined by the manufacturers and in line with established study operating procedures.

HIV testing was conducted onsite with whole blood on the First Response HIV 1+2/syphilis combo card test. This rapid diagnostic test enabled simultaneous detection of antibodies specific to HIV (type 1 & 2) and *Treponema pallidum*. Blood samples reactive for HIV were then subjected to the Oraquick HIV-1/2 test kit. Individuals who were HIV reactive on both First Response and Oraquick were classified as HIV positive. Both HIV and Syphilis results were provided onsite and positive study participants were linked up with a local study partner NGO for referral to an appropriate treatment facility.

At the Regional level, laboratory staff processed blood, and urine samples into 3 aliquots of 1 mL volume per participant. Blood samples were stored frozen at minus 20 degrees Celsius while urine samples were stored in a refrigerator (2 to 8 degree Celsius). On average, it took about a week after the completion of the survey in the Region until the samples in cold boxes were transported to the NMIMR, Accra.

At the NMIMR Virology Department level, study Research Assistants received and transferred the details of blood and urine samples into the study electronic database and stored the blood and urine samples at minus 20 degrees Celsius. All HIV positive samples and 10% randomly selected HIV negative ones were subjected to testing by the INNO-LIA™ HIV I/II Score assay at the NMIMR as a quality assurance measure. All inconclusive HIV results were resolved with this line immunoassay.

All syphilis seropositive samples and 10% randomly selected seronegative ones were subjected to further testing by the Determine Syphilis TP assay as a quality assurance measure. Internal quality control samples were included with each batch of testing. The presence of hepatitis B virus surface antigen (HBsAg) in blood is indicative of an active (either acute or chronic) HBV infection. Testing for HBsAg was conducted with the First Response HBsAg Card Test. All HBsAg seropositive samples and 10% randomly selected seronegative ones were also tested on the Alere Determine HBsAg test as a quality assurance measure. This provided confirmation of hepatitis B virus infection in the study participants.

*Chlamydia trachomatis* (CT) and *Neisseria gonorrhoeae* (NG) Testing: Respondents who reported foul vaginal discharge during the behavioral survey were identified. Their urine samples were tested for CT and NG by the Cepheid Xpert CT/NG Assay, on the GeneXpert® Instrument. The Cepheid Xpert CT/NG real-time PCR test enabled the detection and differentiation of genomic DNA from CT and NG.

## Results

### Demographic characteristics

A total of 7,000 FSW took part in the study with 6,773 (96.8%) taking part in the behavioral interviews while 6,217 (88.8%) took part in the biological component (gave samples for HIV and STI testing). In all, 5,990 (85.6%) of participants completed both the biological and the behavioral components of the study while only 783 (11.2%) and 227 (3.2%) took part in only the behavioral and biological aspects, respectively.

Out of the total of 6,773 (100%) FSW sampled for the behavioral interviews, 42% (2,871) were in the age range of 16-24 years, 49% (3,298) between 25–35 years and about 9% (596) more than 35 years. The median age was 26.5 years old (range 16–68) and seaters were generally older (median age of 28.8). Majority, 84% (5,688) of the FSW were born in Ghana, while 13% (893) reported being born in Nigeria. Nearly 27% of the respondents came from the Greater Accra, Ashanti (19%), Eastern (10%), Western (11%) and Central (9%) regions. The rest of the respondents came from the 11 other regions of the country (0.5 to 6%) as shown in Table 1. About 8% (528) of FSW reported having no formal education, 18% (1,214) completed primary education, and 74% (4,995) completed secondary education and above. Over half, 59% (3,911) of the FSW reported being single / never married while 19% (1263) reported being married. A good percentage of FSW in Western North (89%), Savannah (45%), Western (28%), Northern (21%) and Greater Accra (20%) reported being born in Nigeria.

**Table 1:** Percent distribution of background characteristics of FSW by region, Ghana 2020.

Variables	Western	Central	Greater Accra	Volta	Eastern	Ashanti	Western North	Ahafo	Bono	Bono East	Oti	Northern	Savannah	North East	Upper East	Upper West	(*n*)	(%)
	750	630	1856	173	660	1,271	38	144	411	137	216	171	35	38	174	70	**6,773**	
Age																		
16–24 years	38.4	38.8	38.1	50.1	59.3	37.5	41.6	44.7	42.2	51.9	42.8	51.7	53.7	66.2	49	60.7	2,871	42.4
25–35 years	45.4	55.4	52.4	37.8	36.1	53.9	58.1	54.3	48.2	40.9	44	42.2	44.6	32.6	43	34.1	3,298	48.7
>35 years	16.3	5.7	9.4	12.1	4.6	8.6	0.3	1	9.2	6.2	13.2	6.1	1.1	1.2	6.4	5.2	596	8.8
Non-response	0	0.1	0.1	0	0	0	0	0	0.4	0.9	0	0	0.6	0	1.6	0	8	0.1
Median age in years	27	26	26	24	23	26	25	25	26	24	26	24	24	23	25	23	26.5	
Education																		
No formal education/some primary	6.2	3.3	7.1	7.5	3.2	9.5	2.3	13.1	11.3	15.1	15	11.7	11.4	6.6	12.4	8.5	528	7.8
Completed Primary	21.3	10.9	17.8	27.1	32.1	14.6	4.9	20.6	14.2	14	19.2	11.7	4.9	3.9	15	18.5	1,214	17.9
Some Secondary	72.5	84.5	74.4	65.5	64.6	75.5	92.8	66.2	74.5	69	64.3	75.8	81.7	89.5	72.5	72.3	4,995	73.7
Non-response	0.1	1.3	0.7	0	0.2	0.5	0	0	0	2	1.5	0.8	2	0	0	0.7	36	0.5
Marital status																		
Never married	38.1	42.6	57.2	56.2	70.2	64.7	48.7	52.4	67.9	77.5	43.4	62.9	83.4	82	70.7	70	3,911	58.9
Currently married/cohabiting	29.7	35.6	13	18	11.6	22.1	25.2	22	13.4	9.5	24.6	6.3	1.7	10.4	3.1	5.3	1,263	19.0
Divorced/Separated/Widowed	31.3	21.1	27.3	24.9	17.4	10	25.7	24.6	16.3	10.8	28.9	29.8	12.2	2.5	21.6	22.5	1,458	21.9
Non-response	0.9	0.7	2.6	0.9	0.8	3.2	0.4	1	2.4	2.2	3.1	1	2.7	5.2	4.6	2.3	141	0.2
Religion																		
Christian	83.7	91.2	85.1	83.4	87.1	84.5	95.2	70.3	82.4	73.9	71.1	45.6	77.1	60.2	64.9	34.6	5,570	82.2
Muslim	8.8	5.2	11.4	2.4	8.5	10.2	4.8	19.8	13.9	19.9	18.1	54.4	18.1	38.5	33.1	63.8	870	12.8
Traditional	0.5	0	0.9	4.4	0	1.5	0	0.9	0	0.7	2.2	0	2	0	1	0	56	0.8
No religion	7	3.4	2.3	7.8	4.1	3.7	0	8.9	2	4.5	5.7	0	1.7	1.3	1	1.6	247	3.7
Non-response	0.1	0.3	0.3	2.1	0.3	0.2	0	0	1.7	0.9	3	0	1	0	0	0	30	0.5
Personal income (GHS) from sex work per week	522	383.8	518	330.4	380.7	572.8	344.3	276.4	454.2	462.6	150.6	460.9	725.5	492.3	234.3	379	466	
Country of birth																		
Ghana	70.3	81.4	78.3	79.1	95.6	92.5	2.1	93.8	94.7	96.7	93.2	72.5	50.9	88.4	87.7	94.3	5,688	84
Nigeria	28.4	12.3	19.5	1	3.1	6.8	88.9	6.2	3.6	1.7	1.2	21.3	45.4	5.4	7.4	5.4	893	13.2
Other	1.3	6.3	2.2	19.8	1.3	0.7	9	0	1.6	0.7	5.1	6.2	3.2	6.2	3.9	0.2	185	2.7

There were statistically significant differences between roamers and seaters in terms of age (*p* = 0.006), age at which FSW first sold sex (*p* = 0.004), marital status (*p* = 0.035), consistent condom use with paying clients (*p* = 0.001) and duration of sex work (*p* = 0.001). Seaters were much older, started sex work at later age, divorced/separated or widowed, more consistent in condom use with paying clients, less than 5 years in sex work compared to roamers (Table 2).

**Table 2:** Bivariate analysis between roamers and seaters.

Variables	Roamers	Seaters	Total	χ^2^ (*p*-value)
	4,962	1,747	6,709	
Age				
16–24 years	44.4	32.3	42.4	8.7452 (*p* = 0.006)
25–35 years	48.1	51.0	48.7	
>35 years	7.3	16.6	8.8	
Non-response	0.1	0.1	0.1	
Age first sold sex				
Under 25	76.9	65.0	74.8	8.4336 (*p* = 0.004)
25 to 34	19.3	25.0	20.3	
35 and above	2.1	7.1	2.9	
Non-response	1.7	2.9	2.0	
Education				
No formal/some primary education	7.0	12.6	7.8	1.6579 (*p* = 0.234)
Completed Primary	18.1	17.3	17.9	
Some Secondary	74.4	69.8	73.7	
Non-response	0.6	0.2	0.5	
Marital status				
Never married	59.6	54.9	58.9	1.9524 (*p* = 0.186)
Currently married/cohabiting	19.4	16.9	19.0	
Divorced/Separated/Widowed	20.9	27.7	21.9	
Non-response	0.1	0.4	0.2	
Average number of sexual partners per week				
<11	75.9	67.4	74.5	2.8213 (*p* = 0.101)
11 to 20	15.7	21.3	16.5	
>20	8.2	11.2	8.7	
Non-response	0.3	0.1	0.3	
Consistent condom use with paying clients				
Always used	71.3	77.4	72.2	9.6819 (*p* = 0.001)
Not always used	28.1	22.2	27.1	
Do not know	0.3	0.3	0.3	
Non-response	0.4	0.1	0.4	
Consistent condom use with intimate partner				
Always used	24.3	23.9	24.2	0.7257 (*p* = 0.559)
Not always used	59.4	55.8	58.8	
Do not know	3.5	3.9	3.6	
Non-response	12.8	16.4	13.4	
Comprehensive knowledge about HIV and AIDS				
Yes	34.3	37.2	34.5	1.343 (*p* = 0.315)
No	65.8	62.8	65.5	
Duration of sex work				
< 5 years	54.0	58.5	55.2	29.5725 (*p* = 0.001)
5–10 year	29.4	23.5	27.9	
10–15 years	10.5	10.1	10.4	
15+ years	6.0	7.9	6.5	

### Uptake of HIV services

About three-quarters (74%) of FSW in all sites had ever tested for HIV, with seaters testing more (78.1%) compared to roamers (72.6) and this was statistically significant (*p* = 0.001). Of those who have ever tested, 52.8% had tested within the last 6 months and 23.7% had tested between 7 and 12 months, with seaters (58.7%) testing more than roamers (51.4%). In all, 76.6% of the ever tested (74%) had their test conducted in the last 12 months (Table 3: Uptake of HIV Services by FSW in Ghana Table 3). Furthermore, about 52.8% of FSW had contacts with a peer educator in the past 12 months preceding the survey. Seaters (56.2%) were more likely to have been contacted by peer educator within the last 12 months compared to roamers (52.1%) and this difference was statistically significant (*p* = 0.003). In addition, seaters were more likely to have received condoms and lubricants through outreach (54.6%), counseling on condoms use in the last 3 months (57.8%) compared to roamers with 49.9 and 54.0%, respectively (Table 3).

**Table 3:** Uptake of HIV services by FSW in Ghana.

Variables	Roamers	Seaters	Total	χ^2^ (*p*-value)
	4,962	1,747	6,709	
Ever tested for HIV				
Yes	73.0	78.3	73.8	12.028 (*p* = 0.001)
No	26.7	21.6	25.8	
Do not know	0.1	0.0	0.1	
No response	0.3	0.1	0.3	
Most Recent HIV test				
≤ 6 months	51.4	58.7	52.8	2.4021 (*p* = 0.114)
7–12 months	24.0	23.4	23.8	
> 12 months and ≤ 2 years	13.8	10.1	13.2	
> 2 Years	10.2	7.7	9.7	
Do not know	0.4	0.1	0.3	
Non-response	0.2	0.0	0.2	
Contacts with a peer educator in the last 12 months				
Yes	52.1	56.2	52.8	9.4451 (*p* = 0.003)
No	47.0	43.1	46.2	
Do not know	0.5	0.6	0.5	
Non-response	0.4	0.2	0.4	
Received condoms and lubricants through an outreach				
Yes	49.9	54.6	50.7	31.3713 (*p* < 0.001)
No	49.3	44.7	48.4	
Do not know	0.4	0.3	0.4	
Non-response	0.4	0.4	0.5	
Received counseling on condoms use in the last 3 months				
Yes	54.0	57.8	54.6	5.8778 (*p* = 0.009)
No	45.0	41.7	44.4	
Do not know	0.6	0.4	0.6	
Non-response	0.4	0.1	0.4	
HIV				
Positive	4.5	5.1	4.6	0.6024 (*p* = 0.449)
Negative	95.5	94.9	95.4	
Syphilis				
Positive	1.3	1.4	1.3	0.0522 (*p* = 0.841)
Negative	98.7	98.6	98.7	
Chlamydia				
Positive	5.2	2.3	4.8	2.1828 (*p* = 0.194)
Negative	86.4	93.9	87.5	
Invalid	1.7	0.3	1.5	
Inadequate	6.7	3.5	6.2	
Gonorrhea				
Positive	6.1	3.2	5.7	0.7554 (*p* = 0.378)
Negative	85.5	92.9	86.6	
Invalid	1.7	0.3	1.5	
Inadequate	6.7	3.6	6.2	
Hepatitis B				
Positive	6.9	5.7	6.7	1.5179 (*p* = 0.259)
Negative	93.1	94.3	93.3	

### Sexual history and sex work characteristics

Majority (75%) of FSW first sold sex when they were under 25 years of age, with the median age being 20 years (IQR: 18–25). Most (85%) FSW entered sex work for money for self or family across all the regions. However, for Savannah, a very high proportion (26%) were forced or coerced into sex work. The majority, (68%) were introduced to sex work by a friend and 24% by themselves. Three-quarters (75%) had ten or fewer sex partners per week; the mean number of weekly partners was 9. On average, FSW had eight paid clients in the month before the survey. A much higher proportion of FSW first sold sex while under the age of 25 in Central (83%), Volta (83%), Eastern (88%), Bono (83%), Bono East (90%), and Savannah (90%), compared to the other regions (Table 4).

**Table 4:** Percent distribution of sexual history and sex work characteristics of FSW by region, Ghana 2020.

Variables	Western	Central	Greater Accra	Volta	Eastern	Ashanti	Western North	Ahafo	Bono	Bono East	Oti	Northern	Savannah	North East	Upper East	Upper West	(*n*)	(%)
	750	630	1856	173	660	1,271	38	144	411	137	216	171	35	38	174	70	**6,773**	
Age first sold sex																		
Under 25	61.8	83.2	67.8	83.3	88.1	76.5	62	75.8	82.6	90.4	70.3	76.3	89.5	70.9	77.3	77.1	5,069	74.8
25 to 34	30	15.5	27.5	13.4	9.9	18.7	35.2	22.2	13.7	4.6	20.9	14.1	0	12.6	13.8	16.4	1,377	20.3
35 and above	7.6	1.1	4.1	0.9	1.3	1.6	0.3	0	2.3	2.9	0.5	3.5	0	0	2.1	1.2	195	2.9
Non-response	0.6	0.2	0.7	2.3	0.6	3.2	2.6	2.1	1.4	2.1	8.3	6	10.5	16.5	6.8	5.3	132	2.0
Median age of first sold sex	21	19	22	18	18	20	23	21	19	18	19	20	18	20	18	19	20	
Average age – started sex work	23.2	19.9	22.5	19.1	19.1	20.5	23.1	21.9	19.6	18.8	19.9	21.4	17.7	20.7	19.8	20.4	21.1	
Reasons for entering sex work																		
Money for self/family	85.9	87.8	85.1	91.6	79.6	87	90.6	68.7	85.5	90.5	73.6	92.9	64.6	77.8	78.4	89.4	5,743	84.8
Forced/Pressured/Coerced	2.9	2.9	3.9	3.1	1.2	2.1	9	9.5	2.8	2.8	4.5	2.6	26.2	2.4	1.5	4.2	213	3.1
Pleasure/self-esteem	3.7	2	4.1	2.3	9.3	1.8	0	6.2	4	1.7	6.7	0.3	2.2	10	6.6	0	264	3.9
Friends/Family doing it	4.1	4.4	2.8	1	8.2	2.6	0	10.8	3.9	2.7	9	0.6	3.2	6.5	8.9	1.6	275	4.1
Marital Issues	1.1	1	1.4	1.5	0.2	3.8	0	4.9	1.3	1	2.2	1.6	1.3	1.1	1.6	0.5	119	1.8
Introduced to sex work by																		
Mother	0.6	0.6	0.8	0.1	0.1	0.8	0	0	0.9	1.5	3.7	0.4	5.9	0	2.5	0.9	56	0.8
Father	0	0.8	0.4	0.1	0	0.7	0	1	0.6	0	1.7	0	0.5	0	0.5	0.4	31	0.5
Other relatives	5.7	5.6	2.9	7.6	1.8	5.9	15.7	4.2	2.7	3.7	6.6	4.1	3.2	0	7.8	6.4	300	4.4
Friend	63	63.8	72.2	66.9	67.9	75.4	51.4	55.7	70.9	74.7	31.4	66.8	23.4	76.7	53.3	60.7	4,584	67.7
Nobody	25.6	27.2	20.8	24	29.5	13.8	23.5	37.8	24	17.9	50.5	24.3	64.9	19.7	34.5	25.5	1,605	23.7
Others	1.7	0.9	1.7	0.2	0.5	1.9	9.4	0	0.3	0	0.6	3.5	0	0	1.4	6.1	96	1.4
Non-response	3.4	1	1.2	1.2	0.2	1.5	0	1.3	0.6	2.1	5.5	0.9	2.1	3.6	0	0	100	1.5
Number of sexual partners per week																		
<10	60	77.2	77.8	93.4	93.1	64.3	71.6	76.4	82.0	70.9	97.0	90.9	45.2	85.4	77.9	83.8	4,859	75.8
11 to 20	20.0	14.7	17.3	6.2	6.2	26.7	25.1	23.6	15.5	25.2	3.0	8.3	33.1	7.2	20.0	13.5	1,117	17.5
>20	20.0	8.1	4.9	0.4	0.7	9.0	3.3	0.0	2.5	3.9	0.0	0.8	21.7	7.4	2.1	2.7	423	6.7
Average	12.4	8.6	8.5	5.8	6.3	11.7	10.3	9.3	8.4	10.3	3.7	6.6	16.9	8.4	7.8	6.7	9.0	
Average number paid clients per month																		
One to Two	8.6	13.5	19.6	21.1	32.4	9.4	13.4	2.2	12.1	6.2	53.3	16.8	8.6	9.6	20.3	36.7	1,161	17.1
Three or more	91.4	86.5	80.5	78.9	67.6	90.6	86.6	97.8	87.9	93.8	46.7	83.2	91.5	90.4	79.7	63.3	5,612	82.9
Average number of paid clients	12.5	8.7	7	5.5	4.1	10.4	8.9	6.6	6.5	7.7	2.8	5.7	14.2	10.3	6.7	5.3	7.9	
Main meeting place for clients																		
Sex worker house/rented room/other	42.3	7	20.9	8.3	9.7	8.2	56.1	6.2	12.6	13.6	11.2	10.2	27.2	6.7	17.6	12.1	1,124	16.6
Clients or other homes	6	19.1	6.3	10.1	26	3.1	20.5	23.2	21.5	15	35.8	25.4	41.8	7.8	21.9	17.2	848	12.5
Hotel/Guesthouse	42	72.5	67.3	80.1	58.8	83.3	16.3	66.6	60.7	66.1	47.2	60.3	25.6	80.5	51.9	66.9	4,429	65.4
Pub/Bar/Disco	1.1	0	0.8	1.2	4.1	2.1	4.5	0	3.2	2.5	1.8	0	3.2	3.9	2.1	2.6	109	1.6
Brothel, Massage parlor	8.7	0.6	3.7	0.3	0.6	2	2.6	0	1.4	1	0	3.9	0	0	6.1	1.2	192	2.8

### Condom use

Table 5 describes results for condom use at the national level. Seventy percent (4,781) of FSW in the country reported using condoms consistently (always used condom when having sex within the past 3 months) with a paying client, and 89% (6,029) reported using a condom during the most recent sexual intercourse with a paying client. Majority, 84% (5,115) of FSW reported asking their client to use a condom. Over two thirds (69%) of FSW used condom for HIV and STI prevention while a quarter (23%) reported using condom for both STI and pregnancy prevention. Those who reported not using a condom cited client refusal to use condom (23%), regular client (15%), or paid more (16%) as shown in Table 5.

**Table 5:** Percent distribution of condom use among FSW in Ghana by region, 2020.

Variables	Western	Central	Greater Accra	Volta	Eastern	Ashanti	Western North	Ahafo	Bono	Bono East	Oti	Northern	Savannah	North East	Upper East	Upper West	(*n*)	(%)
	750	630	1856	173	660	1,271	38	144	411	137	216	171	35	38	174	70	**6,773**	
A. Paying clients																		
Consistent condom use	87.5	60.8	83.2	66.3	70.8	70.2	90.5	15.9	52.6	55.7	24.9	77.2	83.1	71.8	52	60.6	4,781	70.6
Condom use with most recent sex with client	96.8	89.4	94.2	84.3	76	92.1	97.1	86.6	87.2	88	46.1	94.9	94.3	95.1	84.2	80.3	6,029	89
Condom use suggested by																		
Client	1.0	3.2	1.4	7.5	4.8	3.2	0.0	4.6	5.6	2.4	20.3	5.1	1.3	1.3	8.7	0.6	196	3.2
Self	93.6	85.3	90.8	86	74.9	92.1	81.1	47.4	65.4	79	49.6	69.8	77.5	64.5	59.9	62.5	5,115	84.2
Mutual Decision	5.2	11.2	7.8	6.4	19.6	4.3	18.9	48	28.1	18.5	30.2	25.1	21.2	34.3	31.5	36.9	746	12.3
The most important reason you use condoms with clients																		
STI/ HIV prevention	70.5	65.2	75.9	85.7	60.2	76	58.9	34.1	72.5	44.8	35.6	58.6	70	49.8	65.8	56.4	4,664	68.9
Pregnancy prevention	0.6	2.7	3.6	5.3	13.6	3.4	4.5	6.5	7	3.9	15.8	9.2	5.4	1.1	4.8	1.5	336	5
Both	26.9	28.8	18.9	6	21.8	19	36.7	57.2	17.8	46.5	20.2	29.5	21.9	46.9	27.1	36.3	1,554	23
Reason for not using condom																		
Client refused	11.5	29.7	15.3	38.3	25.7	13.9	73.2	35	37	31.3	8.7	29.4	56.6	20.7	37.4	13.3	406	23.3
Client is not HIV positive	5.7	3.7	4.6	1.3	10.2	3.8	0	0.1	8.4	19.9	3.3	16.1	0	9.3	0	12.5	95	5.5
Client is a regular client	10.3	19.3	17.1	14.1	14.5	14.5	0	10.3	22.7	19.7	9.8	18.4	0	10.3	10	8.3	268	15.4
I did not have condom	1.7	7.7	4.8	10.5	4.1	8	0	12.7	9.4	2.6	10.2	5.7	11.8	0	18.6	6.1	132	7.6
I want to get pregnant	0	1.8	0	0	0	2	0	0	0.6	1.1	1.8	10.4	0	19.9	0	0	20	1.1
Client paid more	26	8.3	18.2	23	8.3	25	23.7	23.4	12.7	9.8	10.6	6.9	0	0	24.1	6.9	283	16.3
I am afraid of violence from clients	4.3	2.2	0.6	0	0	0	0	8.8	0.5	0	4.5	5.7	0	0	0	1.9	28	1.6
Drunk/ high	3.2	2.1	0.1	0	0	0	0	1.3	0.9	0	2.6	0	0	0	0	0	14	0.8
I do not like condom	11.9	7.1	6.9	10.8	34.4	7.2	0	8.5	2.3	8.1	40.4	0	0	10.3	6.5	31	217	12.5
Had anal sex with client	9.3	11.7	6.5	14.6	5.4	7.5	9.7	44.3	18.7	8.7	17.9	20	0.7	8.9	14	7.9	682	10.1
Condom use with most recent anal sex	63.5	59.6	61.8	62.4	56.5	61.7	76.6	16.3	77.9	93.2	27.2	95.1	100	59.9	56.6	51.8	396	58.4
B. Intimate partner (IP)																		
Consistent condom use	22.6	26.6	17.9	24.6	19.4	28.1	68.6	8.8	28.7	24.7	8.3	34.5	35.5	48	38.7	44.4	1,592	23.5
Condom use with most recent IP	35.9	39.2	27.2	29.5	52.6	49	52	83.8	43.2	57.8	28.3	54	28.1	71.6	58	61.3	2,772	40.9
Reason for not using condom with IP																		
I trusted him	55.9	57.4	62.8	50.5	65.4	80.4	25.2	31.5	72.1	71.1	24.7	35.3	63.9	69	38.1	58.5	2,315	61.8
He refused	16.4	9.1	13.8	32.4	4.9	6.1	16.9	27.1	15.4	9.8	6	12.7	32	5.5	19.6	5.6	431	11.5
He is not HIV positive	10.3	9.9	6.2	0.9	2.5	2.5	2.7	5.8	2.6	6.5	1.4	5.1	0	2.2	1.9	4.2	192	5.1
Did not have condoms	0.3	3.2	0.7	1.7	3.2	0.3	0	9.8	2.2	0.2	1.3	0.8	0	0	18.3	2.4	73	2
I want to get pregnant	0.2	0.9	0.4	0.6	0.2	1.4	0	0	1.8	3.2	0.7	5.7	0	2.7	1.1	0.5	33	0.9
I am afraid he will be violent	3.2	2.3	1.6	0.2	0.3	0.6	1.6	10	0.4	1.2	6.9	0.6	0	0	0	0	65	1.8
I am embarrassed	0.1	0.7	0.3	0	0.3	0	0	0	0	1	1.3	1.4	0	0	0	0	11	0.3
Too drunk/high to use	0.2	0.3	0.6	0.2	0.4	0.2	0	0	0.8	0	0	0	0	0	0.9	0	14	0.4
I do not like it	4.6	4.7	6.5	6.4	6.2	1.7	8.5	11.6	1.2	3.5	45	6.5	0	9.9	8.8	8.6	247	6.6

Consistent condom use with an intimate partner (24%) and condom use at last sex with intimate partners (41%) were much lower compared to condom use with paying clients (71%). The reasons were that FSW trusted their intimate partners (62%) and partners refusal (12%). Much lower rates of consistent condom use with paying clients were found in Ahafo (16%) and Oti (25%). Nearly half (44%) of FSW in Ahafo region reported ever having anal sex, and majority (84%) of them reported condom was not used during the last anal sex act.

### Knowledge of HIV prevention methods

Table 6 shows the distribution of comprehensive knowledge about HIV by background characteristics. Respondents’ knowledge about HIV prevention through the reduction of sexual partners (86%) and consistent condom use (86%) was high. FSW were also asked about common misconceptions regarding AIDS and HIV transmission. Respondents were asked whether they think it is possible for a person to get HIV from mosquito bites, supernatural means such as witchcraft, and for a healthy-looking person to have HIV? While the majority (85%) understood that it is possible for a healthy-looking person to have HIV, 77% knew that HIV could not be transmitted through mosquito bites with just 56% responding correctly that one cannot get HIV from witchcraft. Misconception about HIV and witchcraft was common in Western (38%), Eastern (40%), and Oti (42%). The same pattern of misconceptions was observed in the different regions as well, except for Oti (51%) and North East (68%). They indicated that a healthy-looking person could have HIV. Comprehensive knowledge remains low in Ghana, with only 35% of FSW having comprehensive knowledge with Western (16%) and Oti (13%) regions having the lowest percentages.

**Table 6:** Percent distribution of FSW with comprehensive knowledge about HIV by background characteristics in Ghana 2020.

Background characteristic	Percentage of women who say that:					
	A healthy-looking person can have HIV	Using condoms can reduce chances of getting HIV	Limiting sexual intercourse to one uninfected partner can reduce chances of getting HIV	HIV cannot be transmitted by mosquito bites	HIV cannot be transmitted by supernatural means	Percentage with a comprehensive knowledge about HIV
	6,705	6,721	6,735	6,773	6,714	6,773
Age						
16–24 years	83.1	86.7	86.5	76.3	56.3	32.5
25–35 years	88.1	86.4	86.7	78.3	57.7	37.6
>35 years	84.8	88.7	82.1	68.6	52.7	31.9
Education						
No formal education/some primary	80.1	86.0	88.7	69.7	49.3	25.0
Completed Primary	84.2	86.7	85.8	70.7	48.1	27.4
Some Secondary	86.7	87.0	86.1	78.8	59.5	37.8
Country of birth						
Ghana	85.7	87.1	87.0	77.4	55.3	34.6
Nigeria	86.3	84.5	80.4	71.3	63.2	36.4
Other	88.3	89.8	89.7	76.0	75.5	47.8
Region of residence						
Western	79.7	85.9	84.6	60.2	38.0	15.6
Central	90.6	86.3	88.7	81.0	58.1	36.5
Greater Accra	88.0	84.9	80.4	74.6	57.6	35.1
Volta	94.1	94.3	92.4	62.7	51.0	25.6
Eastern	90.7	90.7	91.4	71.7	40.1	28.7
Ashanti	83.6	88.7	91.3	89.2	65.2	41.3
Western North	87.2	78.8	86.8	72.1	61.9	30.7
Ahafo	100.0	92.6	99.9	98.0	89.2	64.3
Bono	88.0	92.6	88.9	87.2	66.8	47.0
Bono East	97.8	92.3	82.3	76.5	67.6	40.9
Oti	50.5	61.4	71.4	60.6	42.1	12.5
Northern	80.8	84.1	90.2	67.6	73.2	41.2
Savannah	95.7	95.7	73.3	93.9	85.6	67.6
North East	68.6	88.0	66.1	86.4	81.3	44.6
Upper East	85.4	85.9	88.9	75.6	55.4	36.7
Upper West	76.6	89.5	88.8	72.4	66.9	38.4
National	84.9	86.2	85.7	76.6	55.5	34.5

### HIV and STI prevalence

**Figure 1:**
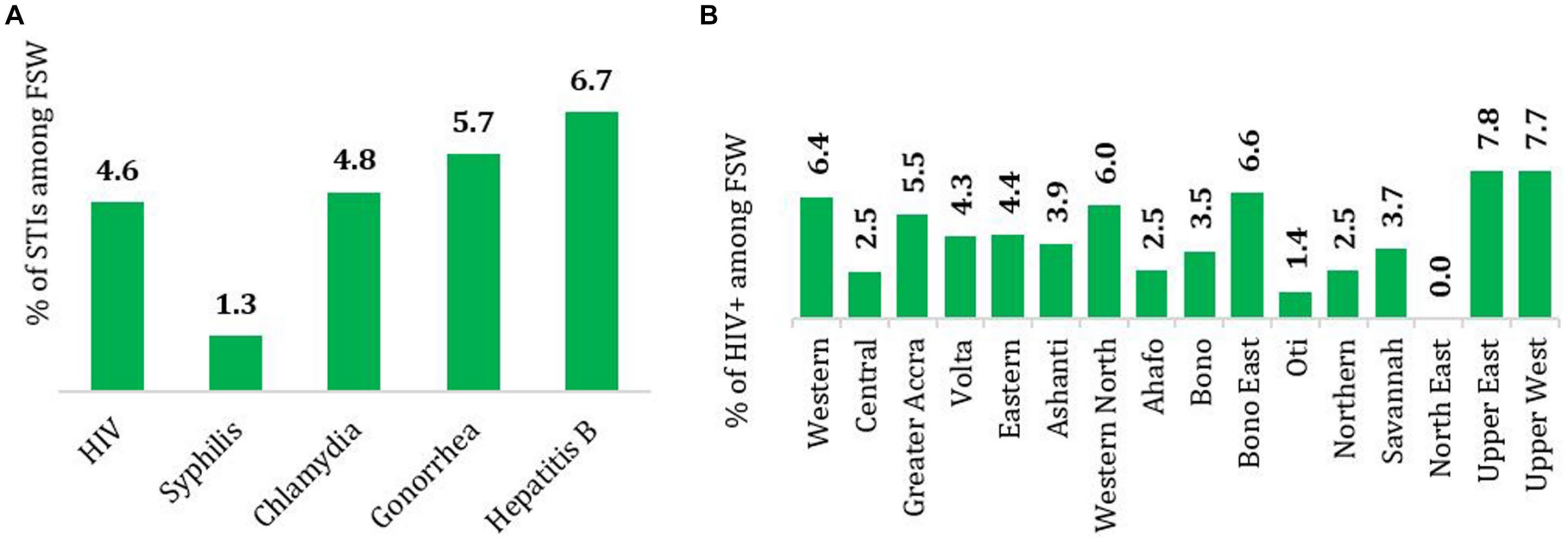
**(A)** National estimates of the prevalence of HIV, syphilis, chlamydia, gonorrhea and hepatitis B and **(B)** prevalence of HIV by region among FSW in Ghana, 2020.

A total of 6,217 FSW participated in the biologic phase of the study. The national HIV prevalence was 4.6%. From the total number of FSW interviewed, only 313 reported having had a foul vaginal discharge, out of which 4.8% tested positive for chlamydia and 5.7% for gonorrhea, Figure 1A. The percentage of FSW who tested positive to syphilis was 1.3%. The highest HIV prevalence was in Upper East (7.8%) and Upper West (7.7%), followed by Bono East (6.6%), Western (6.4%), and Western North (6.0%), Figure 1B. There was no statistically significant relationship between type of FSW (roamer and seater) and HIV status or any of the STIs infections.

**Figure 2:**
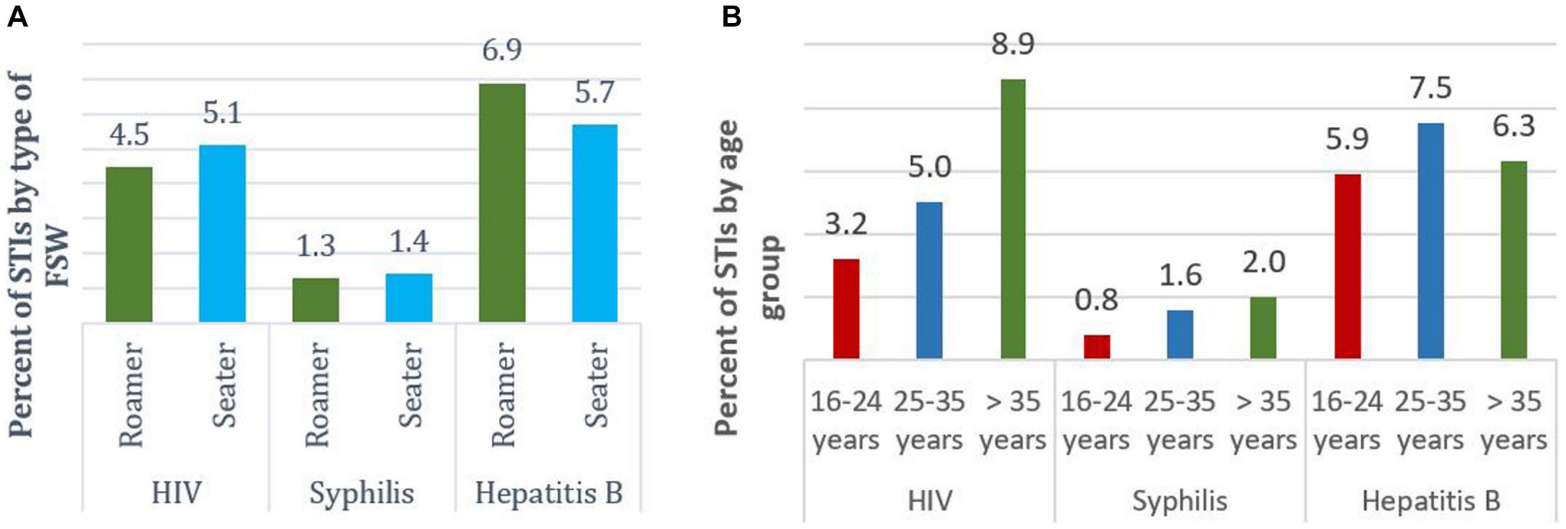
Prevalence of STIs by **(A)** FSW type and **(B)** by Age group among FSW in Ghana, 2020.

HIV prevalence among roamers was 4.5% while that of seaters was 5.1%. There were also variations of prevalence of hepatitis B among roamers and seaters. Observably, hepatitis B prevalence was higher among roamers, Figure 2A. HIV prevalence was higher (8.9%) among the older FSW, that is 35 years and above. The same pattern was observed regarding syphilis. For hepatitis B, the 25-35-year-old group had the highest (7.5%) prevalence, Figure 2B.

### Factors associated with FSW type (seater vs. roamer)

Respondents aged 35 years and above were 2-fold the odds (aOR = 2.0; 95% CI: 1.48, 2.83) of being in the seater community compared to those age < 25 years. There was a higher statistically significant odds of 47% (aOR = 1.47; 95% CI: 1.20, 1.80) and 62% (aOR = 1.62; 95% CI: 1.01, 2.58) among FSW who started working at an age between 25–34 years and 35 years and above, respectively, compared to those who started less than 25 years of being seaters (Table 7).

**Table 7:** Socio-demographic and clinical factors associated with FSW types (seater vs. roamer).

Characteristics	Adj. odds ratio (95% CI)	*p*-value
Age (years)		
<25	ref	
25–35	1.20 (1.00, 1.43)	0.047
> 35	2.05 (1.48, 2.83)	0.000
Age first sold sex		
<25	ref	
25–34	1.47 (1.20, 1.80)	0.000
35 and above	1.62 (1.01, 2.58)	0.045
HIV		
Negative	ref	
Positive	1.14 (0.82, 1.58)	0.431
Average number of sexual partners per week		
<11	ref	
11–20	1.42 (1.17, 1.72)	0.000
>20	1.66 (1.29, 2.13)	0.000
Consistent condom use with paying clients		
Always	ref	
Almost every time	0.79 (0.62, 1.02)	0.066
Sometimes	0.54 (0.42, 0.68)	0.000
Never	0.49 (0.31, 0.77)	0.002
Most recent condom use		
Yes	ref	
No	1.27 (1.09, 1.48)	0.002
Education		
No education	ref	
Completed primary	0.73 (0.54, 0.99)	0.042
Some secondary	0.74 (0.57, 0.97)	0.027

Final adjusted model included only variables that showed public health significance at 5% level. HIV was included in the model priori.

The odds of having between 11–20 clients and above 20 clients in a week were 42% (aOR = 1.42; 95% CI: 1.17, 1.72) and 66% (aOR = 1.66; 95% CI: 1.29, 2.13) higher compared to having less than 11 clients, respectively. The odds of not using condoms during most recent sexual activities were 27% (aOR = 1.27; 95% CI: 1.09, 1.48) higher among seater communities. Among the seater communities, the odds of completing primary and secondary education were 27% (aOR; 0.73 Cl: 0.54–0.99) and 26% (aOR; 0.74 Cl: 0.57–0.97) less likely compared to no education (Table 7).

## Discussion

This survey describes key characteristics and sexual behavior among FSW and estimated HIV and STI prevalence among FSW in all the 10 administrative regions of Ghana. The study highlights the similarities and differences in characteristics of sex workers, sex work, risky behaviors, and HIV/STI prevalence among FSW in Ghana.

Female sex workers were generally young, majority had never been married or were widowed/divorced and a quarter had no education or had only primary education. These findings are similar to what has been reported in the previous BBS (5) and other African countries such as Nigeria (17), Zambia (18), Lesotho (19), and South Sudan (20, 21) where the median age range was between 21 and 28 years, most being single and had primary, secondary or no education. It has been shown from each round of the BBS that several younger females go into sex work. A possible reason for seeing young women involved in sex work could be as a result of trying to cope with the increasing demands for the daily necessities of life or meeting their lifestyle needs. In addition, seaters who maybe divorced/separated or widowed, would go into sex work to meet their economic needs. Further regression analysis show that older FSW are more likely to be seaters than roamers. A study conducted in India on demographic and sex work characteristics of female sex workers found that non-street based FSW get involved with sex work later in life and hence are more likely to be adult women as compared with the street-based FSW (22).

Majority of FSW first sold sex when they were under 25 years of age, with the median age of first selling sex being 20 years. Majority of the FSW who started sex work between the ages of 25–34 and also 35 years and above, joined the seater community, compared to the younger sex workers who were more likely to be roamers. Most of the FSW indicated that they entered sex work for money, either for self or family and had an average of eleven (11) sexual partners per week. This is similar to a study conducted in Eastern Ukraine. They found out that seaters on the average had more clients per week than roamers did. Sex workers who used apartments as their base recorded high (median 35) number of clients within a month than those who are based in art clubs or strip clubs as well as highways and truck-stops (23). Consistent condom use with paying clients was generally unsatisfactory (71%), a decline from 78% of the 2015 BBS. It was, however, very low with their intimate partners or boyfriends (24%) up by 3% of the 2015 BBS (5). Furthermore, only 39% of FSW used a condom in the most recent sex with their intimate partners. The odds of using condoms during their most recent sex activities were less likely among the seaters as compared to the roamers. These findings are consistent with the previous BBS and other studies globally among sex workers, which consistently show low condom usage with intimate partners (24–30). In Nigeria, Okafor et al., conducted a similar study to evaluate the HIV prevalence and high-risk behavior of young brothel and non-brothel based female sex workers. They concluded that brothel-based sex workers reported less condom use with boyfriends and casual partners than non-brothel based. Relatedly, Hao et al., reported that street-based sex workers were more likely to engage in unsafe commercial sex while venue-based were unlikely to use condoms with their regular partners (31). Key reasons cited by the FSW for not using condoms with their intimate partners are trust (accounting for over 60%) and the refusal by the partner to use condom (about 12%). Using trust as a main reason for not using condom with an intimate partner could be worrying, given that most intimate partners also have other partners including some commercial sex partners and sometimes do not use condoms with these partners as well (32, 33). The prominent reasons for not using condom with paying clients include client’s refusal, being a regular client, paying more and FSW indicating that they do not like condoms. These reasons suggest that some FSW focus more on the monetary gains than taking the appropriate steps to reduce the risk of HIV infections and other STIs. The reasons further suggest that power dynamics and environmental factors also influence the sexual behavior of FSW (34). Given that correct and consistent condoms use is one of the most effective measures for reducing HIV transmission among FSW and their partners (35), the consistent low condom use with intimate partners and clients could reverse the progress made in reducing HIV transmission among FSW and their partners. Despite the lack of progress in consistent condom use among non-paying partners, there appear to be no targeted interventions currently implemented for non-paying clients. Specific prevention interventions for non-paying partners should be developed as part of FSW prevention, care and treatment programs while improving on the interventions for the paying clients as well.

These findings show that knowledge of HIV transmission modes is high; majority of FSW know that HIV can be transmitted through sharing injecting equipment, can be passed to an unborn child by an infected mother during pregnancy, delivery or through breastfeeding. However, comprehensive knowledge about HIV (based on knowing that a healthy-looking person could have HIV, using condom consistently and having one uninfected partner can prevent HIV, rejecting the two most common misconceptions that HIV can be transmitted through mosquito bites, and one can get HIV through supernatural means) was very low as only about 35% of FSW have comprehensive knowledge. This result is also consistent with BBS results from Nigeria (17) and Lesotho (19). The main reason for this relatively low level of comprehensive knowledge is the belief that people can get HIV through witchcraft or other supernatural means with significant number of participants holding onto this myth. Given the low level of comprehensive knowledge, there is still the need for targeted education to address strongly held myths and misconceptions about HIV, particularly those surrounding transmission through supernatural means and mosquito bites. Despite the low level of comprehensive knowledge among FSW, it is still relatively higher than among the general population as reported by the 2014 Ghana Demographic and Health Survey (18% among women and 30% among men) (1).

HIV testing has been observed to be the gateway toward prevention, care and treatment. However, there are still a number of FSW who have never tested for HIV and even among those who have ever tested, testing has not been regular as programs aim to test FSW every six months. A number of regions including Ahafo, Western North, Oti, Savannah, North East, Upper East and Upper West have not had any interventions targeting FSW and their intimate partners. These regions have reported low coverage in HIV testing and other prevention activities. There is the need to further expand HIV testing services to all regions to improve testing coverage.

Ghana is making significant progress in reducing the burden of HIV among FSW in the country with the prevalence declining from 11.1% in 2011 to 6.9% in 2015 (5) and to 4.6% in 2020. One of the possible reasons for the decline could be due to the changing population of FSW. Evidence from this study shows that more than half of FSW surveyed were new entrants who had been practicing sex work for less than five years (when the previous BBS was undertaken). Prevalence was higher among those who had been in sex work for more than 10 years, among seaters and older FSW. Despite the general decline, some regions such as Upper East, Upper West, Bono East, Western and Western North have seen significant increase in HIV prevalence. Some of these regions particularly the Upper East and West Regions have not had any interventions targeting key populations for some time now as coverage of HIV services is currently limited in specific geographic locations. The absence of these services in some of the regions might be a factor that is facilitating the high prevalence. Only about half of FSW have been exposed to HIV prevention services in the last three months preceding the survey. The survey shows that some locations without FSW programs are experiencing an increase in HIV prevalence compared to the previous survey. Reaching this target group of new entrants to ensure they access HIV prevention services is critical to reducing the risk of infection.

According to the World Health Organization (WHO), gonorrhoeae is the second most prevalent STI infection globally. In this current study, the prevalence of gonorrhoeae and chlamydia infections are 5.7 and 4.8%, respectively. This finding is similar to other studies previously reported in Kenya among female sex workers in an STI clinic 6.3% (27), women attending an STD referral clinic in Nairobi, 6% (30), 9.1% Isfahan, Iran (32), and 8.3% Yunnan, China (33). The high prevalence of gonorrhoeae and chlamydia as reported elsewhere are as a result of multiple sexual partners and high-risk sexual behaviors such as unprotected sex due to monetary value and alcoholism [229]. This study also estimated the prevalence of hepatitis B to be 6.7% and syphilis to be 1.3%. The prevalence of syphilis among female sex workers in this current study was much lower compared to Ethiopia (6.2%) (31), Nepal (3.9%) (35), Burkina Faso (5.6%) (36), Eastern China (4.31%) (37), and Somaliland (3.1%) (38). Though hepatitis B prevalence is high, it is much lower when compared to Nigeria (17.1%), Ethiopia (9.2%), and Argentina (14.4%) (39–41). Factors such as behavioral, clinical, condom-use, and history of genital ulcer are associated with hepatitis B infection (40).

## Conclusion and recommendations

The survey suggests that FSW in the country are generally young, and majority had never been married or were widowed/divorced, completed secondary education or higher. The findings also give an indication that Ghana is making significant progress in reducing the burden of HIV among FSW in the country. However, the absence of interventions targeting FSW and their intimate partners in some regions, coupled with risky behaviors such as low consistent condom use, and the low coverage of HIV services could reverse the gains made so far. Therefore, the Ghana AIDS Commission and key stakeholders should;

1. Develop different tiers of interventions with a basic or minimum package of service for all sex workers regardless of their locations taking into consideration that more than half of FSW are new entrants. An enhanced or advanced package can then be implemented in all prioritized locations.
2. Develop specific prevention interventions for non-paying partners as part of FSW prevention, care and treatment programs.
3. Come out with targeted education to address the low consistent condom use, and the strongly held myths and misconceptions about HIV, particularly those surrounding transmission through supernatural means and mosquito bites.
4. There is the need to further expand HIV testing utilizing differentiated testing services that include self-testing, social network testing, and enhanced peer outreach.
5. There is the need to develop special HIV interventions targeting older sex workers more especially at the seater communities and carefully target factors that influence unsafe sex among these group of sex workers.

## Data availability statement

The raw data supporting the conclusions of this article will be made available by the authors, without undue reservation.

## Ethics statement

The studies involving humans were approved by University of Ghana Noguchi Memorial Institute of Medical Research Institutional Review Board (CPN 083/18-19), the Ghana Health Service Ethical Review Committee (GHS-ERC 002/05/19) and the Population Council Institutional Review Board (Protocol 891). The studies were conducted in accordance with the local legislation and institutional requirements. The participants provided their written informed consent to participate in this study.

## Author contributions

SD, CG, AA, SA, WA, KA, CA, EK, SW, WT, DA, and KT designed the protocol and supervised all aspects of the study data collection process. KT, CG, and SD analyzed and interpreted the results and wrote the first draft of the manuscript. AA, SA, WA, KA, CA, EK, SW, WT, and DA supported in the final drafting of the manuscript. CG developed and maintained the online database of all the participants’ data, and have full access to all the data in the study and have final responsibility for the decision to submit for publication on behalf of all the contributing authors. All the authors were involved in the training of the data collectors across the country and had access to the data and approved the final version of the manuscript.
